# Biopesticides improve efficiency of the sterile insect technique for controlling mosquito-driven dengue epidemics

**DOI:** 10.1038/s42003-019-0451-1

**Published:** 2019-05-29

**Authors:** David R. J. Pleydell, Jérémy Bouyer

**Affiliations:** 1 CIRAD, INRA, University of Montpellier, UMR ASTRE, F-34398 Montpellier, France; 2 INRA, CIRAD, University of Montpellier, UMR ASTRE, F-97170 Petit Bourg Guadeloupe, France; 3 Insect Pest Control Laboratory, Joint FAO/IAEA Division of Nuclear Techniques in Food and Agriculture, IAEA, Vienna, Austria

**Keywords:** Ecological epidemiology, Computational models

## Abstract

Various mosquito control methods use factory raised males to suppress vector densities. But the efficiency of these methods is currently insufficient to prevent epidemics of arbovirus diseases such as dengue, chikungunya or Zika. Suggestions that the sterile insect technique (SIT) could be “boosted” by applying biopesticides to sterile males remain unquantified. Here, we assess mathematically the gains to SIT for *Aedes* control of either: boosting with the pupicide pyriproxifen (BSIT); or, contaminating mosquitoes at auto-dissemination stations. Thresholds in sterile male release rate and competitiveness are identified, above which mosquitoes are eliminated asymptotically. Boosting reduces these thresholds and aids population destabilisation, even at sub-threshold release rates. No equivalent bifurcation exists in the auto-dissemination sub-model. Analysis suggests that BSIT can reduce by over 95% the total release required to circumvent dengue epidemics compared to SIT. We conclude, BSIT provides a powerful new tool for the integrated management of mosquito borne diseases.

## Abstract (web summary)

David Pleydell and Jeremy Bouyer use mathematical modelling to examine the gains from adding biopesticides to the sterile insect technique for controlling mosquito populations. They find that boosting sterile males with pupicides drastically reduces the total release required to control dengue epidemics.

## Introduction

The international spread of mosquitoes *Aedes aegypti* and *Ae. albopictus* has triggered numerous epidemics of dengue, Zika, chikungunya and yellow-fever^1–4^. Without effective vaccines^5–7^, mosquito abatement remains key to controlling most of these diseases. Mosquito borne pathogens account for one-sixth of infection-associated disability adjusted life years^8^, highlighting the difficulty of area-wide mosquito control^9^. The World Health Organisation has called for new vector control technologies^10^. Here, we explore the potential benefits of combining two prominent *Aedes* control techniques.

The auto-dissemination technique (ADT) uses mosquitoes to deposit biopesticides at larval sites—providing efficient treatment of the small, hidden and disseminated water bodies *Aedes* use as larval habitat^11^. The most common biopesticide used is pyriproxifen—a juvenile hormone analogue inhibiting metamorphosis to adult. Mosquitoes become contaminated with pyriproxifen at dissemination stations^12^. Field trials with pyriproxyfen have demonstrated elevated pupal mortality (emergence inhibition) of 40–70% in *Ae. albopictus* populations^12–15^, and 95–100% density reductions in *Ae. aegypti* populations^16^ and mixed *Ae. aegypti*/*Ae. albopictus* populations^17,18^. Whilst the scale of successful field trials has increased^17,18^, the required high numbers of dissemination stations^19^ impose large maintenance costs and the long-term efficacy of ADT has yet to be demonstrated.

The sterile insect technique (SIT) reduces female reproductive success through sexual competition between wild-type and released males sterilized with ionizing radiation (formerly with chemosterilants)^20,21^. Related methods include the *Wolbachia*-based incompatible insect technique^22,23^, or gene modification systems such as the release of transgenic mosquitoes carrying a dominant lethal^24,25^. Successful SIT programmes have eradicated screwworm and medfly from North and Central America^26,27^, and tsetse from Zanzibar^28^. Mosquito SIT is less developed—while trials have suppressed *Ae. albopictus* populations in Italy^29^, elimination requires maintaining high sterile to wild male ratios which proves prohibitively costly^8^. One proposed solution is to couple SIT and ADT by treating sterile males with biopesticides before release^30,31^. But the efficacy gain from this “boosted” sterile insect technique (BSIT) remains unquantified. Using mathematical modelling, we analyse the efficacy of SIT, BSIT and ADT for controlling *Aedes* vectors and *Aedes* borne diseases.

The dynamics of an *Aedes* population, under BSIT and ADT, were modelled using ordinary differential equations (Eqs. (1)–(8)). The model characterises sexual competition between sterile and wild males^32^, pyriproxifen transfer at dissemination stations^12^, during coupling^30^ and oviposition^11^, and concentration dependent emergence inhibition of juveniles^33^ (Supplementary Fig. 1). Sexual competition depends on the competitiveness (*h*) and relative frequency of sterile males. When pyriproxifen transfer is blocked, the model describes dynamics under standard SIT. Parameterisation (Supplementary Table 1) reflects possible dynamics under fixed favourable climatic conditions across a 1 ha area with 200 larval sites, each of 250 mL and a carrying capacity of 25 larvae. We assume regular maintenance and constant efficacy of dissemination stations, and neglect dispersion^34^, landscape effects^35^, risk-mitigating oviposition site selection^36^, sterile male induced larval site contamination^37^, substrate effects on pyriproxifen efficacy^38^ and reduced female survival due to sexual harassment^39^.

We present results indicating that sexual competition between sterile and wild males creates a threshold sterile male release rate, above which a population density of zero is the only stable equilibrium. Boosting with pyriproxifen generates large reductions in the elimination threshold, the sub-threshold stable equilibrium, the total number of sterile males required for elimination, and the time to elimination. An equivalent elimination threshold does not exist for the auto-dissemination technique, which is most efficient at large densities. Epidemiological analyses suggest if using SIT, without pyriproxifen and with near elimination threshold release rates, the equilibrium density of female mosquitoes can be greater than the density of females required to bring the basic reproductive number of dengue below one. This suggests that vector elimination may be required to prevent dengue epidemics—something that has yet to be achieved with mosquito SIT (or related techniques). Boosting with pyriproxifen lowers both the elimination threshold and the stable equilibrium, providing greater protection against dengue, possibly even if elimination is not achieved. We conclude that ADT and SIT are complimentary techniques and that BSIT can provide a powerful new approach for protecting populations against diseases such as dengue, chikungunya and Zika.

## Results

### Boosting reduces the thresholds and time for elimination

**Fig. 1:**
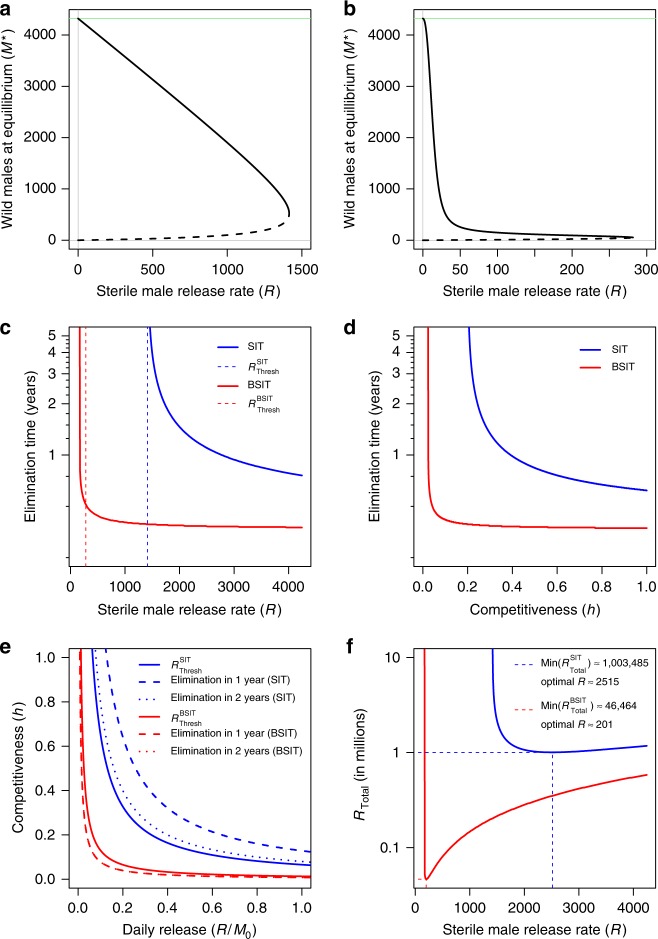
Equilibria, thresholds and optima of the BSIT model. Density of males at stable (solid lines) and unstable (dashed lines) equilibria given release rate (*R*) under SIT (**a**) and BSIT (**b**). A bifurcation, where stable and unstable equilibria converge, provides an elimination threshold for SIT $\left( {R_{{\mathrm{Thresh}}}^{{\mathrm{SIT}}} = 1414.7} \right)$. Pyriproxifen reduces the threshold $\left( {R_{{\mathrm{Thresh}}}^{{\mathrm{BSIT}}} = 281.6} \right)$ and the distance between stable and unstable equilibria (**b**). With SIT, elimination time grows asymptotically at $R_{{\mathrm{Thresh}}}^{{\mathrm{SIT}}}$ (blue dashed), whereas boosting can shift this asymptote below $R_{{\mathrm{Thresh}}}^{{\mathrm{BSIT}}}$ (red dashed) (**c**). With *R* fixed (*R* = 1414), elimination time responds asymptotically to competitiveness (**d**), and boosting shifts the threshold (*h*_Thresh_) towards zero. Thresholds for the eventual elimination of any initial population (solid), and for eliminating from carrying capacity in one (dashed) or two (dotted) years respond non-linearly to release rate (*R*) and competitiveness (*h*) (**e**). Two years (dotted) and 1 year (dashed) elimination thresholds for BSIT (red) are indistinguishable. The total release required for elimination (*R*_Total_) is minimised at $1.8 \times R_{{\mathrm{Thresh}}}^{{\mathrm{SIT}}}$ and $0.7 \times R_{{\mathrm{Thresh}}}^{{\mathrm{BSIT}}}$ for SIT and BSIT, respectively (**f**, dashed lines). All simulations were initialised at carrying capacity, with *M*_0_ the initial density of males

With SIT, augmenting the daily release rate (*R*) decreases the asymptotic population density (stable equilibrium) (Fig. 1a, Supplementary Fig. 2A). A bifurcation, where stable and unstable equilibria converge, gives a threshold release rate $R_{{\mathrm{Thresh}}}^{{\mathrm{SIT}}}$. Maintaining $R > R_{{\mathrm{Thresh}}}^{{\mathrm{SIT}}}$ ensures eventual elimination, whilst $R < R_{{\mathrm{Thresh}}}^{{\mathrm{SIT}}}$ ensures convergence to a new stable equilibrium (Supplementary Figs. 2A, B). Elimination times rise asymptotically at $R_{{\mathrm{Thresh}}}^{{\mathrm{SIT}}}$ (Fig. 1c) and quasi-zero gradients near $R_{{\mathrm{Thresh}}}^{{\mathrm{SIT}}}$ can trap trajectories for many years (Supplementary Fig. 2B). Thus, *Aedes* elimination with SIT requires $R \gg R_{{\mathrm{Thresh}}}^{{\mathrm{SIT}}}$ and sustaining such high release rates entails non-trivial logistic difficulties^29,40^.

Boosting reduces the bifurcation point (by 80%) and the distances between stable and unstable equilibria (Fig. 1b). Unlike SIT, for BSIT the elimination time asymptote shifts to some $R < R_{{\mathrm{Thresh}}}^{{\mathrm{BSIT}}}$ (Fig. 1c). The size of this shift depends on initial population densities—large populations generate high pyriproxifen concentration peaks (Supplementary Fig. 2C). When these peaks push populations beneath the unstable equilibrium elimination becomes easy, otherwise transient oscillations and population recovery lead to a new stable equilibrium (Supplementary Fig. 2D).

Elimination time responds asymptotically to sterile male competitiveness *h* at a threshold *h*_Thresh_. With *R* = 1414, boosting reduces *h*_Thresh_ by 80% (Fig. 1d). Sensitivity analyses suggest boosting induced reductions in *h*_Thresh_ would be greatest for low *R*—but even with daily release rates as high as the adult male carrying capacity (*M*_0_) boosting could reduce *h*_Thresh_ by as much as one order of magnitude (Supplementary Fig. 3A). Thresholds *h*_Thresh_ and *R*_Thresh_ are highly sensitive to variation in near-threshold values of *R* and *h*, respectively (Supplementary Figs. 3A, B). Elimination with sub-threshold values of *R* (or *h*) requires sufficient pyriproxifen accumulation to prevent population recovery to a new stable equilibrium (Supplementary Figs. 3C, D). With *R* fixed at 500, an upper bound on *h*_Thresh_ is sensitive to egg viability *q*, whereas the expected value of *h*_Thresh_ shows greatest sensitivity to the quantity of pyriproxifen deposited at oviposition (*p*) and its longevity in the environment (1/*d*) (Supplementary Fig. 4A). Similar patterns are observed with *R*_Thresh_ (Supplementary Fig. 4B) and (with *R* = 1500) elimination time and *R*_Total_ (Supplementary Fig. 4C, D). The elimination thresholds *R*_Thresh_ and *h*_Thresh_ are interdependent and boosting permits rapid elimination under many *R* ~ *h* combinations that would only suppress mosquitoes under SIT (Fig. 1e).

Control practitioners need to identify release rates that can eliminate vectors with a minimum of sterile males. Numerical integration indicates that, to eradicate a population initialised at carrying capacity, SIT requires at least 1,003,485 sterile males released over 399 days, while BSIT requires just 46,464 sterile males released over 231 days (Fig. 1f)—an efficiency gain of over 95%. These results suggest that BSIT may achieve elimination in many scenarios where it is impractical with SIT.

### Boosting shrinks the basic reproductive number of dengue

**Fig. 2:**
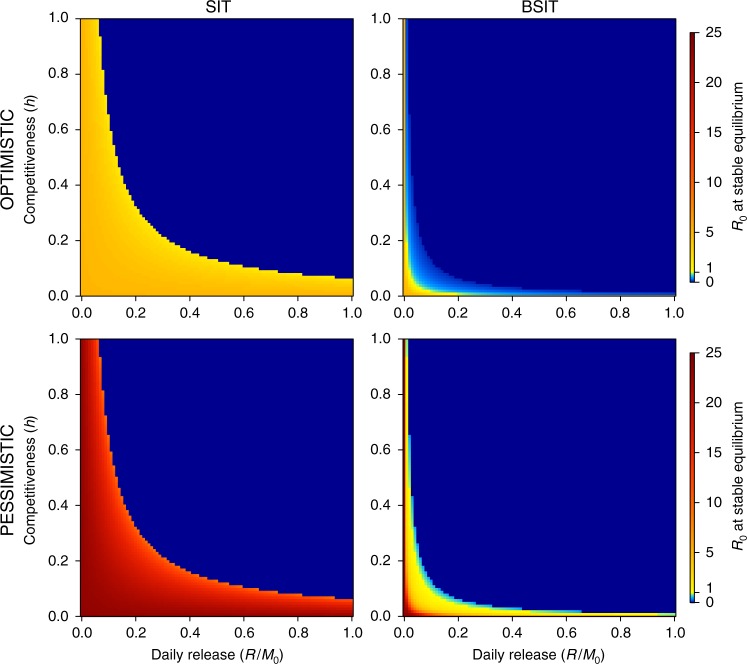
Basic reproductive number (*R*_0_) of dengue transmission. Shown as a function of sterile male competitiveness (*h*) and release rate (*R*) for SIT (left) and BSIT (right). Alternative “optimistic” (top row) and “pessimistic” (bottom row) parameter sets are used (Supplementary Table 2). *M*_0_ is the control-free stable equilibrium of males

To assess the epidemiological implications of boosting, the BSIT model was coupled with a dengue transmission model where transmission occurs between susceptible, exposed, infectious or recovered humans and susceptible, exposed or infectious female mosquitoes^41^ (Supplementary Fig. 5, Eqs. (19)–(28)). For simplicity, we neglect spatial dynamics^42,43^, temperature driven parameter fluctuations^41,44^, inapparent infections^45,46^, non-linearity in bite rates^47–49^, multiple serotypes^50–52^ and multi-annual cyclicity^50,53^. Parameters (Supplementary Table 2) reflect transmission within 1 ha accommodating 50 susceptible humans. This small spatial scale was adopted to minimise bias from assuming homogeneous mixing and to characterise transmission at localised hot-spots with high vector-host ratios^54,55^. The asymptotic stable equilibrium of the system was used to calculate the basic reproductive number (*R*_0_)—recall, epidemic spread in a susceptible population requires *R*_0_ > 1. *R*_0_ calculation used two parameter sets, labelled “optimistic” and “pessimistic”, with different bite rates, transmission probabilities and extrinsic incubation periods (Supplementary Table 2). The notation $R_0^{{\mathrm{Opt}}}$ and $R_0^{{\mathrm{Pes}}}$ indicates the *R*_0_ associated with each parameter set. Boosting reduced *R*_0_ for many combinations of *R* and *h* and expanded the region of parameter space over which *R*_0_ < 1 (Fig. 2). For SIT, the relation between *R*, *h* and the *R*_0_ unity threshold (Fig. 2) matched the elimination thresholds (Fig. 1e). For BSIT, some *R* ~ *h* combinations lead to $R_0^{{\mathrm{Opt}}} < 1$ and $R_0^{{\mathrm{Pes}}} < 1$ (light blue) without vector elimination (dark blue). More *R* ~ *h* combinations were associated with $R_0^{{\mathrm{Opt}}} < 1$ than with $R_0^{{\mathrm{Pes}}} < 1$. Thus, BSIT (but not SIT) might provide lasting protection against dengue without the need for elimination, particularly in situations where a more optimistic parameterization of *R*_0_ is justifiable.

### Auto-dissemination is most effective at high densities

**Fig. 3:**
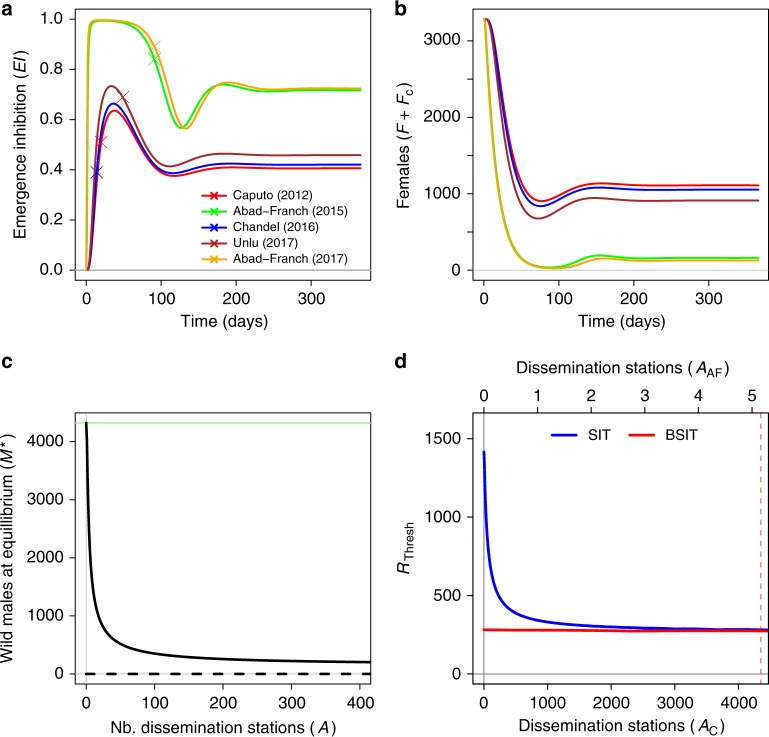
Trajectories, equilibria and thresholds with ADT. Trajectories of emergence inhibition under ADT, and calibration points taken from five field trials (**a**). Corresponding trajectories of total female density (**b**). Stable (solid) and unstable (dashed) equilibria of the ADT model as a funciton of dissemination station density (**c**). The response of the elimination thresholds $R_{{\mathrm{Thresh}}}^{{\mathrm{SIT}}}$ (blue) and $R_{{\mathrm{Thresh}}}^{{\mathrm{BSIT}}}$ (red) to dissemination station density (**d**), where *A*_C_ indicates dissemination station density when contamination rate *α* is estimated from Caputo et al.^12^, and where *A*_AF_ indicates dissemination station density when *α* is estimated from Abad-Franch et al.^18^ (Supplementary Table 3). The vertical pink dashed line indicates the dissemination station density required for $R_{{\mathrm{Thresh}}}^{{\mathrm{SIT}}}$ to equal $R_{{\mathrm{Thresh}}}^{{\mathrm{BSIT}}}$ with *A* = 0 (intercept of red line)

To assess whether ADT could augment SIT and BSIT efficacy, we estimated the contamination rate at dissemination stations (*α*) using emergence inhibition (*EI*) data from five field trials (Supplementary Table 3). Estimates of *α* were greatest for trials targeting mixed *Ae. albopictus*/*Ae. aegypi* populations (Supplementary Table 3). Some authors have suggested ADT is more efficient when *Ae. aegypi* is present^17^, and our analyses are consistent with that hypothesis. All *EI* trajectories peaked rapidly and then oscillated to convergence at a stable equilibrium (Fig. 3a). An inverse pattern was observed in female density, where an initial crash was followed by recovery to a stable equilibrium (Fig. 3b). The stable and unstable equilibria of the ADT sub-model do not converge when dissemination station density (*A*) is increased (Fig. 3c). Without a bifurcation, zero remains an unstable equilibrium, suggesting it would be highly unlikely to eliminate *Aedes* using ADT alone. Even if the total contamination rate (*α* × *A*) was high, low mosquito numbers would not sustain sufficient *EI* to prevent recovery.

### Auto-dissemination improves SIT and BSIT efficacy

The elimination threshold of BSIT $(R_{{\mathrm{Thresh}}}^{{\mathrm{BSIT}}})$ is reduced by ADT—but the reduction is extremely small (Fig. 3d, red line). For SIT, the effect of ADT on $R_{{\mathrm{Thresh}}}^{{\mathrm{SIT}}}$ is greater (Fig. 3d, blue line). The size of this reduction depends on the total contamination rate *α* × *A* —we call this rate the ADT “intensity” for brevity. With *α* based on Caputo et al.^12^ data (*α* = 0.0035), it would require *A* = 4350 dissemination station per hectare for $R_{{\mathrm{Thresh}}}^{{\mathrm{SIT}}}$ to match $R_{{\mathrm{Thresh}}}^{{\mathrm{BSIT}}}$ without ADT (Fig. 3d, pink dashed). This number drops three orders of magnitude using *α* estimated from Abad-Franch et al.^18^ (Fig. 3d, top axis).

**Fig. 4:**
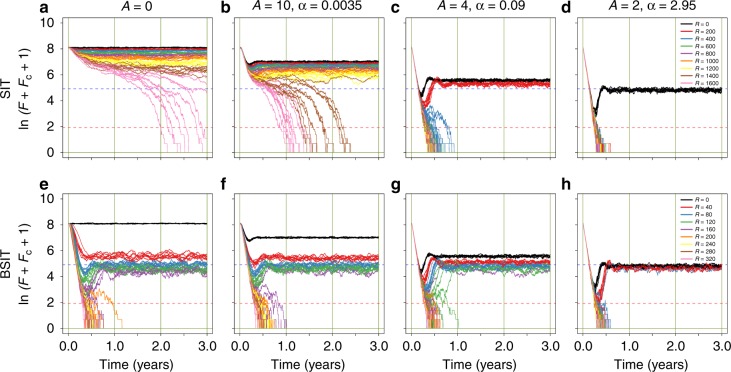
Stochastic simulation of *Aedes* control with SIT, BSIT and/or ADT. Mosquito populations were initiated as Poisson random variables with expectancies set to the control-free stable equilibrium. For SIT (top row) and BSIT (bottom row), nine different release rates were evaluated (see colour legends). For ADT, four different intensities (*α* × *A*) were evaluated. Values of *α* in columns two to four were estimated from different field trials (Supplementary Table 3), the associated values of *A* were adjusted to provide a more even spread of intensities. For each *R* ~ *A* combination, ten simulations are shown. Total female density (plus one) is shown on the natural log scale. Thresholds in female density corresponding to $R_0^{{\mathrm{Opt}}} = 1$ and $R_0^{{\mathrm{Pes}}} = 1$ are indicated as blue and pink dashed lines, respectively. In all simulations, an immigration rate of zero was assumed

To account for small-population effects, we complimented the deterministic analyses above with stochastic simulation^56,57^. Mosquitoes (initialised at carrying capacity) were subjected to SIT or BSIT with ADT applied at four different intensity levels. With BSIT, trajectories either displayed transitory oscillations followed by convergence to a (stochastic) stable equilibrium, or destabilisation followed by elimination (Fig. 4). With *R* = 0, ADT displayed similar transitory dynamics, but with a higher stable equilibrium and no elimination (black lines). For SIT and *A* = 0, elimination was only achieved when $R > R_{{\mathrm{Thresh}}}^{{\mathrm{SIT}}}$ (Fig. 4a)—the minimum time to elimination was over 2 years, reflecting that $R - R_{{\mathrm{Thresh}}}^{{\mathrm{SIT}}}$ was too small for rapid elimination. Increasing ADT intensity lowered the stable equilibria and increased the probability and rate of elimination. At highest ADT intensity, SIT achieved elimination with *R* as low as 200, and trajectories resembled those of BSIT with *R* close to $R_{{\mathrm{Thresh}}}^{{\mathrm{BSIT}}}$ (Fig. 4d, e). When *A* = 0, the (non-zero) stable equilibria were lower for BSIT than for SIT. Increasing ADT intensity reduced this difference. For BSIT, only trajectories leading to elimination sustained $R_0^{{\mathrm{Pes}}} < 1$, but several trajectories converged below the $R_0^{{\mathrm{Opt}}}$ unity threshold. For SIT, only trajectories leading to elimination sustained $R_0^{{\mathrm{Opt}}} < 1$. Without ADT, boosting reduced by one order of magnitude the release rates at which the probability of elimination became non-negligible, and elimination was faster with boosting. Using ADT alone (*R* = 0), even the highest intensity ADT scheme did not suppress mosquito densities sufficiently to sustain $R_0^{{\mathrm{Opt}}} < 1$. These results suggest boosting can provide a greater level of protection against dengue than would be possible with SIT or ADT alone. Moreover, an ADT-SIT combination could only provide the same level of protection as BSIT with either highly efficient (*α*) or highly numerous (*A*) dissemination stations.

## Discussion

At present, insect control primarily depends on insecticides, with major impacts on human/animal health and food safety^58^. Moreover, negative effects of chemicals on predator populations, and the evolution of insecticide resistance, can trigger outbreaks of target (or secondary pest) populations and control failure^59^. Various mosquito release schemes (SIT, incompatible insect technique, transgenic mosquitoes) are being tested in the hope of establishing more efficient control without the undesirable impacts of insecticides. Yet despite the sophistication of modern methods, we remain incapable of preventing large-scale epidemics of mosquito borne diseases. Our analyses highlight a tight association between release rate and competitiveness thresholds which provide minimum conditions for elimination with SIT. Boosting with pyriproxifen shifts these thresholds, and could reduce by over 95% (Fig. 1f) the number of sterile males required for *Aedes* elimination.

Auto-dissemination field trials have reported impressive levels of suppression^17,18^. However, our analyses suggest several potential problems with ADT: a lack of bifurcation makes elimination difficult; ADT works well at high, but not low, mosquito densities; some degree of population recovery is expected once pyriproxifen levels fall; very high *EI* has yet to be demonstrated over prolonged periods, or in the absence of *Ae. aegypti*. Coupling ADT with SIT or BSIT therefore makes sense. These methods work best at low densities and introduce a bifurcation that renders zero a stable equilibrium. Thus, so long as release rates greater than *R*_Thresh_ are maintained, population recovery can be kept in check.

Our model is relatively simple and relies on laboratory data for the dose–response curve^33^ and venereal pyriproxifen transfer^11^. Using data from alternative emergence-inhibition studies had little impact on the difference between $R_{{\mathrm{Thresh}}}^{{\mathrm{SIT}}}$ and $R_{{\mathrm{Thresh}}}^{{\mathrm{BSIT}}}$. Although our estimate of female induced pyriproxifen transfer (*p*) relies on one key study^11^, by neglecting direct male induced contamination of larval sites we likely underestimate pyriproxifen accumulation^37^—particularly at low female densities. The magnitude of pupicidal action in our model corresponds well with field-cage experiments and field trials that measured the impacts of pyriproxifen transfer from (non-sterilised) males to females and larval sites^37^. Moreover, whilst control trials with transgenic mosquito OX513A in Brazil and the Cayman Islands have demonstrated suppression of *Ae. aegypti* populations, in both cases the release area was reduced mid-trial to augment *R* locally^40,60^, and elimination with transgenic mosquitoes has never been demonstrated^8^. Similarly, field trials with the incompatible insect technique have only demonstrated suppression, not elimination, resulting in the up-scaling of insect production^61^. Our analyses are consistent with these observations, and explain why (Fig. 1e) sustaining sufficient release rates for *Ae. aegypti* elimination appears unrealistic given the competitiveness (*h* < 0.06^40^) of OX513A. Boosting offers a powerful solution to the pragmatic and economic difficulties of *Aedes* elimination with released male methods.

Historically, *Ae. aegypti* has been the vector primarily associated with dengue. Some authors have questioned whether confounding factors, such as historical geographical distribution, have led us to underestimate the vector competence of *Ae. albopictus*^62^. The traditional view was that epidemics associated with *Ae. albopictus* were small—such as those in Tokyo^55^, Guangdong (before 2014)^63^ and Arunachal Pradesh^64^. However, this view has been challenged by large outbreaks on Reunion Island (over 8500 indigenous cases in 2018–2019^65^) and in Guangdong (over 45,000 indigenous cases in 2014^66^). The auto-dissemination technique has been shown to provide *EI* > 90% in the presence of *Ae. aegypti*, apparently with sufficient suppression to achieve *R*_0_ < 1^18^. However, the same level of suppression has yet to be demonstrated where *Ae. albopictus* is the sole vector. Our results suggest that BSIT can provide greater protection from dengue than is possible using ADT or SIT alone.

Whether or not mosquito abatement achieves *R*_0_ < 1 depends upon numerous factors. We calculated *R*_0_ using two different parameters sets reflecting variation in the literature of some key parameters. Whether or not those parameters are appropriate in a given control scenario will depend upon the specifics of the local ecology. We neglect several sources of complexity such as temperature effects^67,68^, variation in the availability of alternative hosts^69,70^, dispersion^34^ and landscape effects^35^. We do this for simplicity and emphasise that care is required when extrapolating our results to real systems. Further simulation studies accounting for seasonality and spatial heterogeneity in mosquito ecology would be beneficial for generalising our results to real control scenarios.

One highly variable *R*_0_ parameter is the vector-host ratio—but estimating the density of an insect that is a master of stealth is difficult. Vector densities can be highly aggregated in space^71^ and localised hot-spots play an important epidemiological role^55^. Without detailed knowledge of local vector densities, *R*_0_ studies rely on assumptions such as simply assuming the ratio is two^49^, or that trap density and true density are equivalent^18^. Sophisticated density estimation studies have used mark-release-recapture with BG-Sentinel traps and modern spatial statistics^72,73^—although these studies often report much lower densities than those given by human landing collections. In Forli district, Italy, human landing collections in August averaged 5.73 females (s.d. = 4.48) in 15 min^54^. Fitting a negative binomial distribution to that data suggests 5% of sampling sites provided over 14 females per human-trap in 15 min—numbers well within the range of findings from a park in Tokyo^55^. Assuming the majority of biting females within a 7 m radius are sampled within 15 min, extrapolating over 1 ha and using the bite rate used in that study (0.258), gives a mean of $\frac{{5.73 \times 100^2}}{{0.258 \times \pi \times 7^2}} = 1442.7$ females/ha and a 95th percentile of $\frac{{14 \times 100^2}}{{0.258 \times \pi \times 7^2}} = 3525.0$ females/ha. This later estimate is similar to the carrying capacity of our study (*F* = 3283.9)—thus our ecological assumptions are coherent with known hot-spots in Italy. On Reunion Island, densities of over 5800 males/ha have been reported using mark-release-recapture with mice-baited BG-Sentinel traps^74^—our modelling predicts that, if using SIT alone, it would be very difficult to bring *R*_0_ below one with such high mosquito densities.

The current study has focused on control within 1 ha. Given that *Aedes* densities display spatial auto-correlation over just some hundreds of metres^71^, 1 ha would be a suitable pixel size for an *R*_0_ mapping study^75,76^ in an urban area. Although we have not modelled spatial effects, it is important to remember spatial processes when interpreting our results. Bringing *R*_0_ below one locally may have near-zero impact on the epidemiology across a large city^77^. Also, whilst BSIT might be able to achieve elimination with $R < R_{{\mathrm{Thresh}}}^{{\mathrm{BSIT}}}$, this phenomenon relies on high mosquito densities generating a large pyriproxifen peak. Subsequent immigration would facilitate population recovery unless *R* was greater than $R_{{\mathrm{Thresh}}}^{{\mathrm{BSIT}}}$, or some higher threshold if using a ADT-SIT combination without boosting. Thus an area-wide vector management strategy is recommended, and spatial simulation models, extending the current model by including crucial sources of ecological variation, are expected to provide valuable information for planning mosquito control.

Whilst extreme weather events can reduce pyriproxifen efficacy^78^, large ADT trials in towns of the Amazonian rain-forest suggest regular rainfall does not prevent population suppression^17,18^. In our model, we neglect this potential source of variation. We also do not include variation in container size, thus heterogeneity in pyriproxifen concentrations is neglected. Whilst water tanks and tires are known to provide good larval habitat for *Aedes*, large bodies of water, such as stagnant swimming pools, appear to be less important—particularly when they receive little shade^79^. Moreover, large non-cryptic habitats are relatively easy to identify and treat manually (with pyriproxifen granules, for example ref. ^80^). The greatest difficulty faced by traditional methods of *Aedes* control comes from the high aptitude of these mosquitoes to utilise small cryptic habitats that are protected from insecticide spraying. It is here that both SIT and ADT excel, both techniques utilise mosquito behaviour to bypass the limits of conventional spraying methods. Moreover, where bigger pools are attractive to *Aedes*, they will attract more pyriproxifen carrying mosquitoes, thus increasing pyriproxifen accumulation. Further research is required to fully understand the effects of container size and climate in ADT and BSIT field trials.

Pyriproxifen is highly toxic for all water invertebrates, thus care should be taken regarding undesirable ecological impacts of its use. However, with BSIT or ADT, pyriproxifen contaminated females are expected to specifically contaminate their larval habitats. In urban areas, 95% of *Ae. albopictus* breeding habitats are domestic containers and 99% are of artificial type^81^—factors which should limit the risks for non-target fauna. Thus, the environmental risks are expected to be much lower than those of the widely used technique of ultra-low volume spraying^78^. However, it is important to monitor the impacts of ADT and BSIT on non-target organisms when testing in field conditions—a factor that has been overlooked in many field trials to date.

In light of the large effects predicted here—and the coherence between model results and available field trial data—we urge mosquito control practitioners/developers to include BSIT in their field trials to further quantify its potential. Although we have concentrated on pyriproxifen, alternative biopesticides could be used. Densoviruses, for example, may advantageously provide: greater species specificity; replication at larval sites, ensuring efficacy even with low transfer rates; and an additional tool for resistance management^31^. Given the aptitude of *Aedes* mosquitoes for range expansion, the high burden of associated epidemics, and the resilience of *R*_0_ to modest declines in vector density, the results of such trials would be of great importance for global health management.

## Methods

### Boosted sterile insect technique model

The dynamics of an *Aedes* population in response to the sterile insect technique (SIT), boosted sterile insect technique (BSIT) and/or auto-dissemination technique (ADT) were modelled using the following system of ordinary differential equations.

1 $$E{\prime} = (F + qF_c)gf\frac{M}{{M + hS}} - E(m_E + \mu _E),$$

2 $$L\prime = Em_E - L\left( {m_L + \mu _0 + \frac{{\mu _K - \mu _0}}{K}L} \right),$$

3 $$P{\prime} = Lm_L - P(m_P + \mu _P),$$

4 $$F{\prime} = P\rho m_P\left( {1 + \left( {\frac{{C/V}}{{EI_{50}}}} \right)^\sigma } \right)^{ - 1} + F_c\frac{\gamma }{{\kappa _c}} - F\left( {\frac{{rhS}}{{F + F_c}} + \alpha A + \mu _F} \right),$$

5 $$M{\prime} = P(1 - \rho )m_P\left( {1 + \left( {\frac{{C/V}}{{EI_{50}}}} \right)^\sigma } \right)^{ - 1} - M\mu _M,$$

6 $$S{\prime} = R - S\mu _S,$$

7 $$F{\prime}_c = F\left( {\frac{{rhS}}{{F + F_c}} + \alpha A} \right) - F_c\left( {\frac{\gamma }{{\kappa _c}} + \mu _c} \right),$$

8 $$C{\prime} = F_c\gamma p - Cd.$$

Compartments in this system include eggs (*E*), larvae (*L*), pupae (*P*), adult females (*F*), adult males (*M*), adult sterile males (*S*), pyriproxifen carrying (contaminated) adult females (*F*_*c*_) and the quantity of pyriproxifen at larval sites (*C*). Parameters (Supplementary Table 1) include daily release rate (*R*); dissemination station density (*A*); gonotrophic cycle rate (*g*); female fecundity per gonotrophic cycle (*f* ); maturation rates of juveniles (*m*_*E*_, *m*_*L*_, *m*_*P*_); mortality rates of eggs (*μ*_*E*_), pupae (*μ*_*P*_), females (*μ*_*F*_), contaminated females (*μ*_*c*_), males (*μ*_*M*_) and sterile males (*μ*_*S*_), larval mortality—a linear function of larval density rising from *μ*_0_ at *L* = 0 to *μ*_*K*_ at *L* = *K* (carrying capacity); the proportion of females among emerging adults (*ρ*); the number of larval sites (*N*); the volume of water at larval sites, *V* = *V*_1_ × *N*, where *V*_1_ is the mean volume per site; the carrying capacity at larval sites, *K* = *K*_1_ × *N*, where *K*_1_ is the mean carrying capacity per site; the contamination level (in parts per billion, ppb) generating 50% emergence inhibition (*EI*_50_); the slope of the dose–response curve modelling emergence inhibition among maturing juveniles (*σ*); the mating rate of wild males (*r*); competitiveness of sterile males (*h*); the mating rate of sterile males (*rh*); viability of eggs from contaminated females (*q*); oviposition rate (*γ*); expected number of ovipositions per gonotrophic cycle (*κ*); number of ovipositions required to clear contamination (*κ*_*c*_) and the expected quantity of pyriproxifen deposited by contaminated females at oviposition (*p*).

The term $\frac{M}{{M + hS}}$ is a classic representation of sexual competition^32^, providing the proportion of couplings involving wild-type males (Eq. (1)). Sterilisation is assumed absolute. The competitiveness of sterile males, *h*, is the ratio of (per capita) sterile male to wild male coupling rates. The strength of sexual competition depends on *h* and the relative frequency of *M* and *S*—represented as two red dashed arrows in Supplementary Fig. 1. Based on Gaugler et al.^11^, we assume adult females become contaminated when they couple with males carrying pyriproxifen. These events occur at rate $\frac{{rhS}}{{F + F_c}}$ per female (Supplementary Fig. 1, red dashed arrows). These females deposit *p* µg of pyriproxifen at larval sites according to oviposition rate (*γ*) and lose their pyriproxifen after an expected *κ*_*c*_ ovipositions—we assume *κ*_*c*_ = 1 throughout. The term $\left( {1 + \left( {\frac{{C/V}}{{EI_{50}}}} \right)^\sigma } \right)^{ - 1}$ provides the emergence success according to a logit(*EI*) ~ ln(*C*/*V*) dose–response curve (Supplementary Fig. 1, red dashed arrows). Swapping the logit function for probit, and/or the natural logarithm for log_10_, made little difference to the dose–response curve—hence, we adopted the algebraically and computationally more convenient form. Parameter *σ* gives the slope of a straight line on the transformed scales. The model assumes pyriproxifen degrades in the environment at constant rate *d*.

### Equilibria analysis

Differential Eqs. (1)–(8) return gradients of zero at (respectively)

9 $$E^ \ast = \frac{{(F + qF_c)gfM}}{{(M + hS)(m_E + \mu _E)}},$$

10 $$L^ \ast = \frac{{ - (m_L + \mu _0) \pm \sqrt {(m_L + \mu _0)^2 + 4Em_E(\mu _K - \mu _0)/K} }}{{2(\mu _K - \mu _0)/K}},$$

11 $$P^ \ast = \frac{{Lm_L}}{{(m_P + \mu _P)}},$$

12 $$F^ \ast = \frac{{ - {\cal{B}} + \sqrt {{\cal{B}}^2 - 4{\cal{A}}{\cal{C}}} }}{{2{\cal{A}}}},$$

13 $$M^ \ast = Pm_P(1 - \rho )\left( {1 + \left( {\frac{{C/V}}{{EI_{50}}}} \right)^\sigma } \right)^{ - 1}/\mu _M,$$

14 $$S^ \ast = R/\mu _S,$$

15 $$F_c^ \ast = \frac{{ - {\cal{E}} + \sqrt {{\cal{E}}^2 - 4{\cal{D}}{\cal{F}}} }}{{2{\cal{D}}}},$$

16 $$C^ \ast = \frac{{F_c\gamma p}}{d},$$

where * indicates the values at which the respective ordinary differential equations have zero gradient and

$$\begin{array}{l}{\cal{A}} = \alpha A + \mu _F,\\ {\cal{B}} = {\cal{A}}F_c + rhS + {\cal{C}}/F_c,\\ {\cal{C}} = - F_c\left( {P\rho m_p\left( {1 + \left( {\frac{{C/V}}{{EI_{50}}}} \right)^\sigma } \right)^{ - 1} + F_c\frac{\gamma }{{\kappa _c}}} \right),\\ {\cal{D}} = \frac{\gamma }{\kappa }_c + \mu _c,\\ {\cal{E}} = F({\cal{D}} - \alpha A),\\ {\cal{F}} = - F(\alpha AF + rhS).\end{array}$$

A trivial equilibrium of the system exists at $E^ \ast = L^ \ast = P^ \ast = M^ \ast = F^ \ast = F_c^ \ast = C^ \ast = 0$ and S* = *R*/*μ*_*S*_. Non-trivial equilibria of the system are found (for a given *R*) at the intersections of the following two curves describing M^*^ as a function of *F*. With *R* and *F* fixed, S*, $F_c^ \ast$ and C* are obtained from Eqs. (14)–(16). Then, assuming *F*′ = *F′*_*c*_ = *M′* = 0, a process of substitution using Eqs. (4) and (7) gives

17 $$M^ \ast = (F\mu _F + F_c^ \ast \mu _c)(1 - \rho )/(\mu _M\rho ).$$

Secondly, assuming *E*′ = *L*′ = *P*′  = *M*′ = *S*′ = *F*′_*c*_ = *C*′ = 0, we obtain

18 $$M^ \ast = \frac{{ - {\cal{H}} + \sqrt {{\cal{H}}^2 - 4{\cal{G}}{\cal{I}}} }}{{2{\cal{G}}}},$$

where

$$\begin{array}{l}{\cal{G}} = \mu _M/\left( {m_P(1 - \rho )\left( {1 + \left( {\frac{{C^ \ast /V}}{{EI_{50}}}} \right)^\sigma } \right)^{ - 1}} \right),\\ {\cal{H}} = hS^ \ast + \frac{{(m_L + \mu _0) \ast K \ast m_L}}{{(\mu _K - \mu _0)(m_p + \mu _p)}},\\ {\cal{I}} = \frac{{Km_L}}{{(\mu _K - \mu _0)(m_P + \mu _P)}}\left( {(m_L + \mu _0)hS^ \ast - \frac{{(F + qF_c^ \ast )gfm_Lm_E}}{{{\cal{G}}(m_E + \mu _E)(m_P + \mu _P)}}} \right).\end{array}$$

When *R* < *R*_Thresh_, the curves (17) and (18) intersect at two points, giving one stable equilibrium and one unstable equilibrium. When *R* = *R*_Thresh_, the curves meet at a single point. When *R* > *R*_Thresh_, the curves no longer intersect and the population will eventually be eliminated—irrespective of the initial population density. The equilibria can be found using standard root finding algorithms.

### Parameterisation of BSIT model

Parameters were set to values obtained from the literature (Supplementary Table 1). Shape parameter *σ* of the dose–response curve was estimated from published *EI*_50_ and *EI*_95_ data^33^ as the slope of the straight line linking these two data points on transformed (logit(*EI*) ~ ln(*C*/*V*)) scales. The quantity of pyriproxifen deposited by a female at oviposition (*p*) was estimated by using the dose–response curve to predict the concentration of pyriproxifen in the water of the venereal transfer experiments of Gaugler et al.^11^ based on their reported emergence inhibition. The quantity deposited per oviposition (*p*) was obtained assuming contaminated females lose their pyriproxifen in a single oviposition. The obtained value of *p* was then divided by five to account for Gaugler et al. using five males to one female in their venereal transfer experiment. Using alternative emergence inhibition data to generate the dose–response curve had relatively little impact on our modelling results—$R_{{\mathrm{Thresh}}}^{{\mathrm{BSIT}}}$ was estimated as 286.1, 253.9, 299.0, 174.9 and 328.2 using emergence inhibition data from refs. ^33,82–84^ and ref. ^85^ (Rockefeller strain), respectively. The relative viability of eggs from contaminated females (*q*) was assessed experimentally^86^. Two to five-day-old fertile males were sprayed with a dry powder containing 20% pyriproxifen and mated with 5-day-old virgin females. Egg papers were dried for 24 h and emergence was monitored for 8 days. The expected value of *q* and bootstrap 95% confidence intervals are shown in Supplementary Table 1.

In our model, each female mosquito is contaminated at ADT dissemination stations with rate *α* × *A*—for brevity we call this rate the ADT “intensity”. Field trial data (Supplementary Table 3) provided five different estimates of *α*. For each trial we identified the *α* that minimised the absolute error between reported and fitted *EI* at a given point in time. We assumed *EI* at larval sites matched *EI* in ovitraps. Minimisation was performed over a finite sequence of 100 evenly spaced values spanning two orders of magnitude. The appropriateness of the bounds of this finite set were checked visually by plotting the absolute error for each potential *α*. Since our model is deliberately simple, we did not expect it to characterise the full range of *EI* variation observed in the field. Therefore, we did not explore more complicated methods (such as Bayesian methods^87,88^) for fitting mechanistic models. Regarding uncertainty in *α*, we note that the estimates are highly variable between studies, and that an estimate from any one study might not transfer well to other ecological contexts.

### Time to elimination

The time required to bring the total population size below one, when initialised at carrying capacity (the control-free stable equilibrium), was evaluated using R function ode^89^ and is called “elimination time” throughout the paper. All such simulations used a constant sterile male daily release rate *R*. The quantity *R*_Total_ was defined as the product of elimination time and *R*.

### Sensitivity analyses

Eight sensitivity analyses with the BSIT/SIT model were performed (Supplementary Figs. 3 and 4). Parameters were sampled uniformly over the plotted ranges, all other parameters were set to default values (Supplementary Table 1). Each experiment consisted of 10^5^ randomisations of the selected parameter set. Trends in the generated data clouds were explored using the R function loess^90^.

### Dengue transmission model

The epidemiological model of dengue transmission was adapted from ref. ^41^ by splitting compartment *F* into *F*_*S*_, *F*_*E*_ and *F*_*I*_ and compartment *F*_*c*_ into $F_{c_S}$, $F_{c_E}$ and $F_{c_I}$. The model uses Eqs. (1)–(3), (5)–(6) and (8) (where *F* and *F*_*c*_ are the sum of their respective sub-compartments) and the following sub-system:

19 $$F{\prime}_S = P\rho m_P\left( {1 + \left( {\frac{{C/V}}{{EI_{50}}}} \right)^\sigma } \right)^{ - 1} + F_{c_S}\frac{\gamma }{{\kappa _c}} - F_S\left( {b\beta _F\frac{{H_I}}{{H_\Sigma }} + \frac{{rhS}}{{F_\Sigma }} + \alpha A + \mu _F} \right)$$

20 $$F{\prime}_E = F_Sb\beta _F\frac{{H_I}}{{H_\Sigma }} + F_{c_E}\frac{\gamma }{{\kappa _c}} - F_E\left( {\theta _F + \frac{{rhS}}{{F_\Sigma }} + \alpha A + \mu _F} \right)$$

21 $$F{\prime}_I = F_E\theta _F + F_{c_I}\frac{\gamma }{{\kappa _c}} - F_I\left( {\frac{{rhS}}{{F_\Sigma }} + \alpha A + \mu _F} \right)$$

22 $$F{\prime}_{c_S} = F_{_S}\left( {\frac{{rhS}}{{F_\Sigma }} + \alpha A} \right) - F_{c_S}\left( {b\beta _F\frac{{H_I}}{{H_\Sigma }} + \frac{\gamma }{{\kappa _c}} + \mu _c} \right)$$

23 $$F{\prime}_{c_E} = F_{c_S}b\beta _F\frac{{H_I}}{{H_\Sigma }} + F_E\left( {\frac{{rhS}}{{F_\Sigma }} + \alpha A} \right) - F_{c_E}\left( {\theta _F + \frac{\gamma }{{\kappa _c}} + \mu _c} \right)$$

24 $$F{\prime}_{c_I} = F_{c_E}\theta _F + F_I\left( {\frac{{rhS}}{{F_\Sigma }} + \alpha A} \right) - F_{c_I}\left( {\frac{\gamma }{{\kappa _c}} + \mu _c} \right)$$

25 $$H{\prime}_S = \mu _H(H_\Sigma - H_S) - (F_I + F_{c_I})b\beta _H\frac{{H_S}}{{H_\Sigma }}$$

26 $$H{\prime}_E = b\beta _H(F_I + F_{c_I})\frac{{H_S}}{{H_\Sigma }} - (\theta _H + \mu _H)H_E$$

27 $$H{\prime}_I = H_E\theta _H - (\alpha _H + \mu _H)H_I$$

28 $$H{\prime}_R = \alpha _HH_I - H_R\mu _H$$

where $F_\Sigma = (F_S + F_E + F_I) + (F_{c_S} + F_{c_E} + F_{c_I})$ is the total population density of adult females, $H_\Sigma = H_S + H_E + H_I + H_R$ is the total population density of humans, *b* is the bite rate of a single female, *β*_*F*_ is the probability that a susceptible female mosquito becomes infected having bitten an infectious human, *β*_*H*_ is the probability that a susceptible human becomes infected following a bite from an infectious mosquito, *θ*_*F*_ and *θ*_*H*_ are (respectively) the extrinsic and intrinsic incubation rates and *α*_*H*_ is the recovery rate in humans.

### The basic reproductive number of dengue transmission

The basic reproductive number (*R*_0_) was calculated using the next generation matrix approach^91,92^. Assuming the mortality rate of females carrying pyriproxifen (*μ*_*c*_) equals that of females without pyriproxifen (*μ*_*F*_) permits *R*_0_ to be written

29 $$R_0 = \sqrt {\frac{{(F_S + F_{c_S})}}{{H_\Sigma }}b\beta _F\frac{{\theta _F}}{{(\theta _F + \mu _F)}}\frac{1}{{(\alpha _H + \mu _H)}}b\beta _H\frac{{\theta _H}}{{(\theta _H + \mu _H)}}\frac{1}{{\mu _F}}} .$$

Throughout, we assume $H_\Sigma = 50$ people/ha, a population density typical of many European cities (such as Montpellier or Seville). Two alternative parameterizations were used (Supplementary Table 3), reflecting variation in the literature and providing “optimistic” and “pessimistic” estimates of *R*_0_.

### Stochastic simulation of population dynamics under control

To incorporate the effects of demographic stochasticity in small populations, and the non-equilibrium dynamics in the first months of control, stochastic simulation (of integer events) was performed using a modification of Gillespie’s direct algorithm^56^. To reduce computation time we incorporated modifications presented in ref. ^57^ and adopted the following two approximations: eggs were generated in batches per oviposition event with batch size set as either a draw from a Poisson distribution (when *E* < 5000) or the expected number of new eggs (at higher densities); egg maturation and mortality was simulated using either the tau-leap method (when *E* < 5000)^93^ or by using the solution to the linear ordinary differential equations (at higher densities). The algorithm was coded in Nimble^94^, which automatically compiles code with R-like syntax to C++. Scripts for all analyses are available. For SIT and BSIT, nine values of *R* were evenly spaced in the intervals [0, 1600] and [0, 320], respectively. ADT was applied at four levels of intensity corresponding to either no ADT, or ADT with intensity equivalent to Caputo et al.^12^, Abad-Franch et al.^17^ with *A* reduced from 14 to 4 or Abad-Franch et al.^18^ with *A* increased from 1.54 to 2. The values of *α* and *A* used in each scenario are shown in Fig. 4. Ten simulations were performed for each *R* ~ *A* combination. In each simulation, mosquitoes were initialised by drawing Poisson random numbers with expectancies given by the control-free stable equilibrium, and control parameters were held constant for 3 years.

### Reporting summary

Further information on research design is available in the Nature Research Reporting Summary linked to this article.

## Supplementary information

Supplemental Material

Reporting Summary

## Data Availability

Data sharing not applicable to this article as no datasets were generated or analysed during the current study.

## Code availability

All code used in the current study is available at the following Bitbucket repository https://DRJP@bitbucket.org/DRJP/pleydell-bouyer-2019.git.
