# Supplementary material for: Biopesticides improve efficiency of the sterile insect technique for controlling mosquito-driven dengue epidemics
Source: Commun Biol. 2019 May 29;2:201. doi: 10.1038/s42003-019-0451-1 (PMC6541632; doi:10.1038/s42003-019-0451-1)
Supplement: Supplementary file 1 — Supplemental Material [file 42003_2019_451_MOESM1_ESM.pdf]

Supplementary Figures

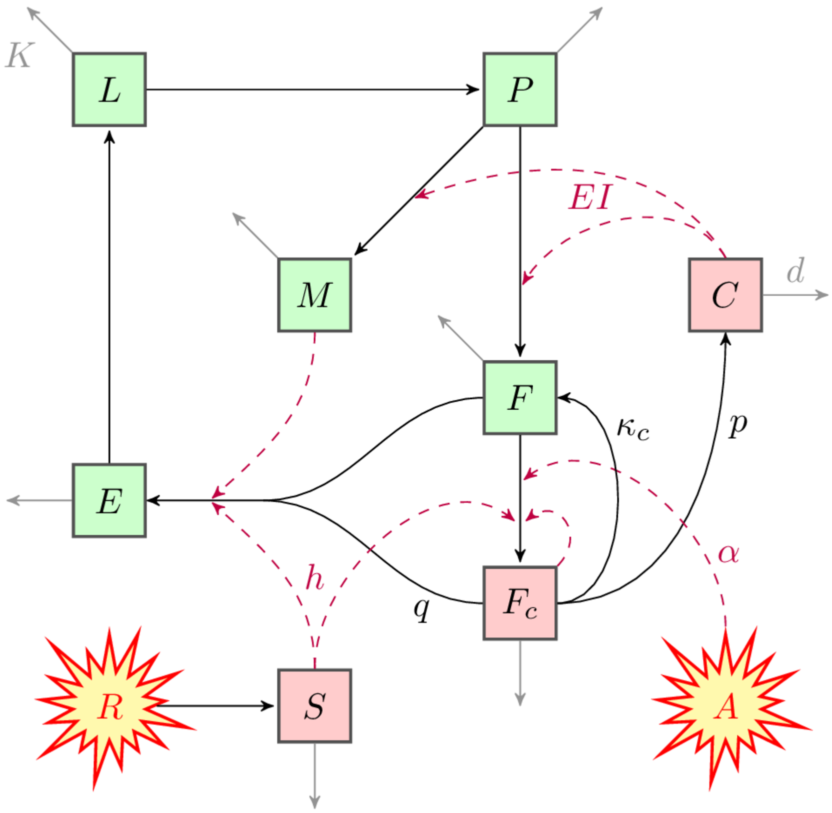

Supplementary Figure 1: Graphical representation of the SIT/BSIT/ADT *Aedes* control model. The life-cycle (green boxes) consists of egg (E), larvae (L), pupae (P) and adult males (M) and females (F). Control elements (red boxes) include pyriproxifen-coated sterile males (S), contaminated females (F_c) and the concentration of pyriproxifen at larval sites (C) which determines emergence inhibition (EI). Total population size is regulated by larval carrying capacity (K). Control parameters include: sterile male release rate (R); density of dissemination stations (A); sexual competitiveness (h); the quantity of pyriproxifen deposited at oviposition (p); egg-viability of contaminated females (q); the rate for one dissemination station to contaminate a single female (α); and the larval site decontamination rate (d). Female decontamination is expected after κ_c ovipositions. Red dashed arrows indicate effects on transition rates (black arrows). Constant female fecundity (f), gonotrophic cycle rate (g) and stage specific maturation (m) and mortality (μ) rates are assumed (not show).

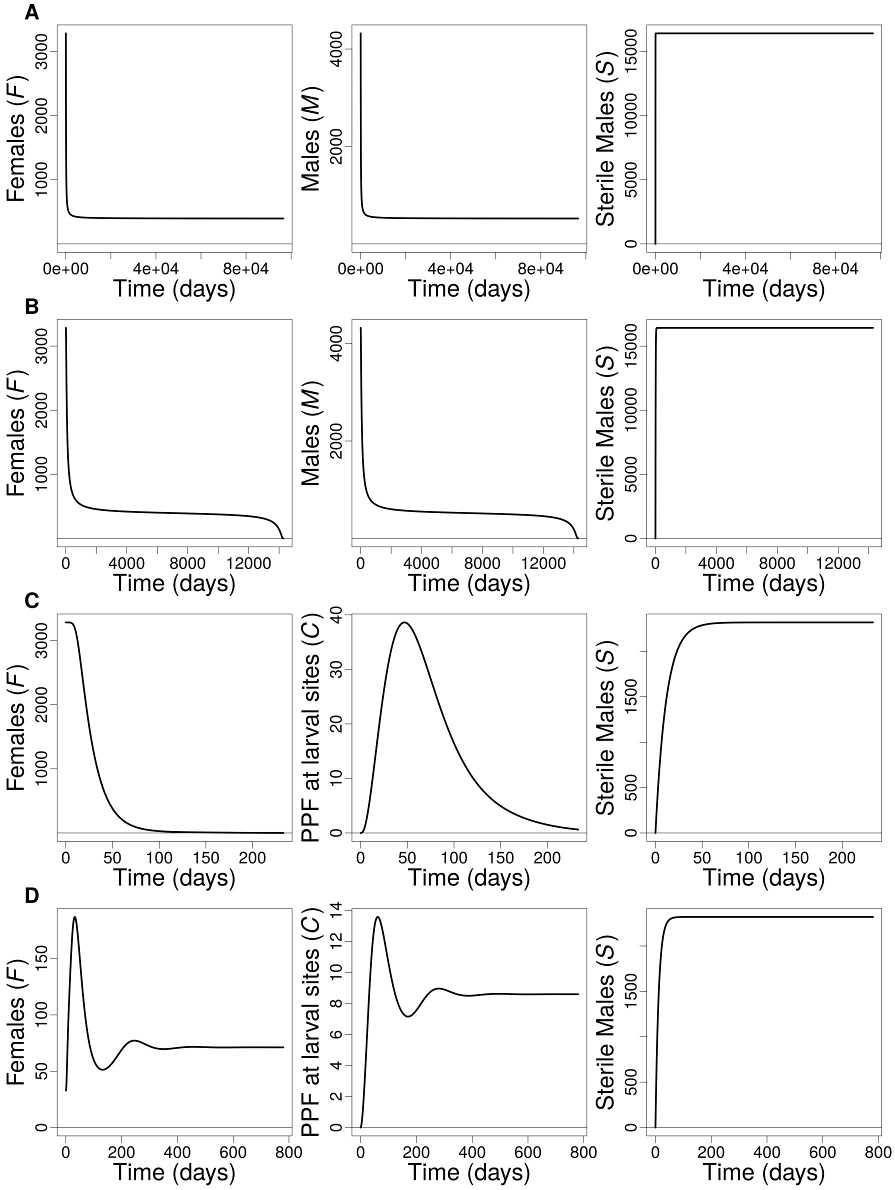

Supplementary Figure 2: Trajectories of the SIT/BSIT model under four different parameterisations. For SIT, when release rate R is less than elimination threshold $R_{\text{Thresh}}^{\text{SIT}}$ ($R = 1414$) densities converge to a stable equilibrium (A). With R slightly greater than $R_{\text{Thresh}}^{\text{SIT}}$ ($R = 1415.7$) densities become trapped at a pseudo-equilibrium that delays elimination (B). Conversely, elimination with BSIT can be possible when $R < R_{\text{Thresh}}^{\text{BSIT}}$ (here $R = 200$) provided sufficient quantities of pyriproxifen (PPF) accumulate at larval sites (C). When initial population densities are low (set here at 1% of the control-free stable equilibrium) trajectories of C exhibit transient oscillations and the population converges to a stable equilibrium (D). Increasing the initial population increases the amplitude of the transient oscillations and when the initial peak in C is sufficiently large the system destabilises and elimination becomes possible (C).

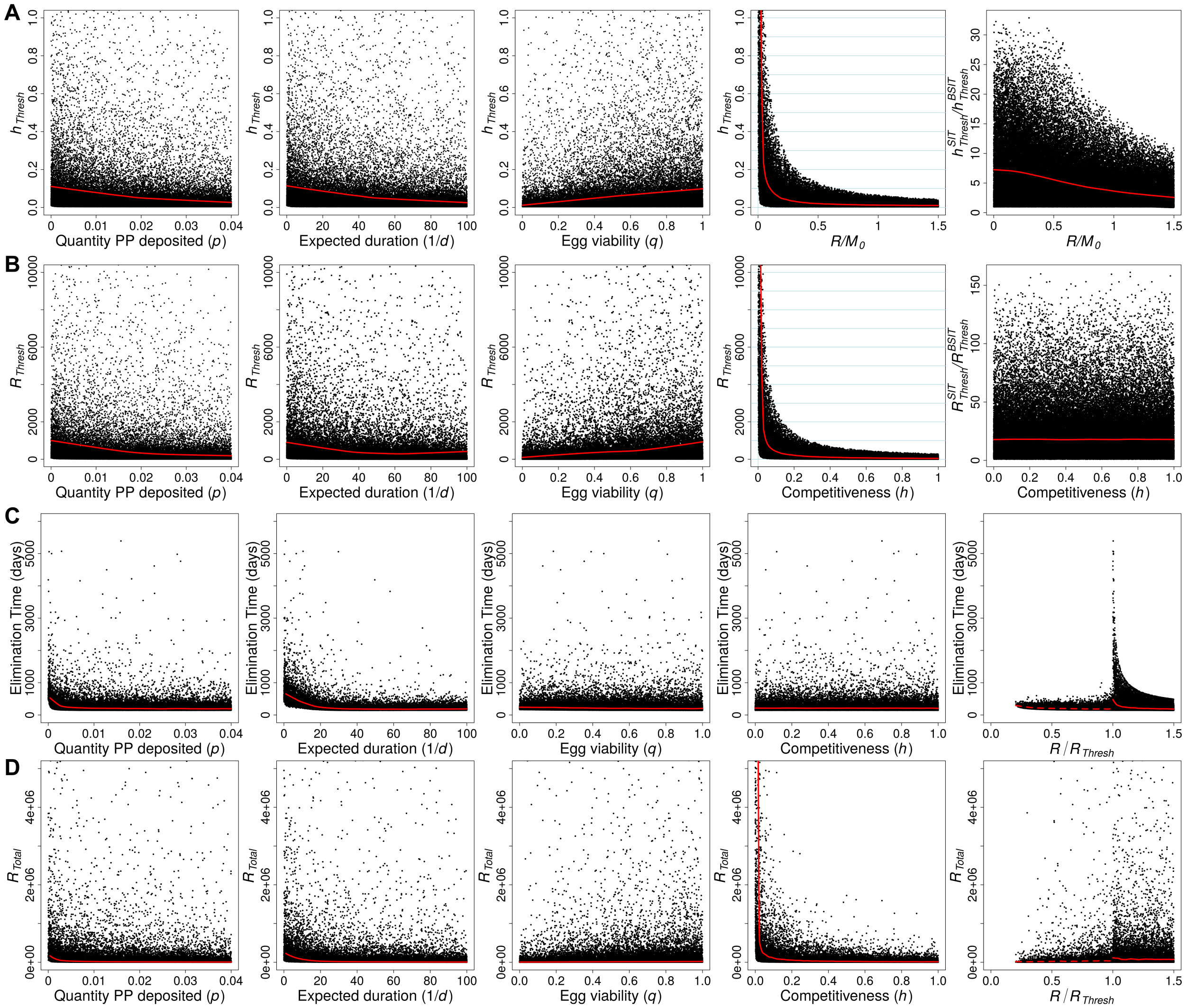

Supplementary Figure 3: Sensitivity analysis of BSIT model. Sensitivity of h_{Thresh} (A), R_{Thresh} (B), elimination time (C) and total release for elimination (D) to control parameters in the BSIT model. The threshold h_{Thresh} is most sensitive to release rate R (shown relative to the carrying capacity of males M_0) and the gain from boosting $h^{\text{SIT}}_{\text{Thresh}}/h^{\text{BSIT}}_{\text{Thresh}}$ is greatest for low R (A). The threshold R_{Thresh} is most sensitive to sterile male competitiveness (B). Elimination time responds differently depending on whether R is greater than or less than R_{Thresh}: when $R > R_{\text{Thresh}}$ the probability of elimination is one and the mean (solid) and variance in elimination time become large close to R_{Thresh} (C); when $R < R_{\text{Thresh}}$ rapid elimination is possible given sufficient accumulation of pyriproxifen (dashed), otherwise a new stable equilibrium is obtained and elimination time becomes infinite (not shown). The total release for elimination (R_{Total}) is most sensitive to competitiveness h – as $h \to 0$ neither SIT nor BSIT can have any effect (D). In all simulations (C and D), the population was initialised at the control-free asymptotic equilibrium. One hundred thousand parameter randomisations are used per row. Rows C and D use the same set of parameter randomisations.

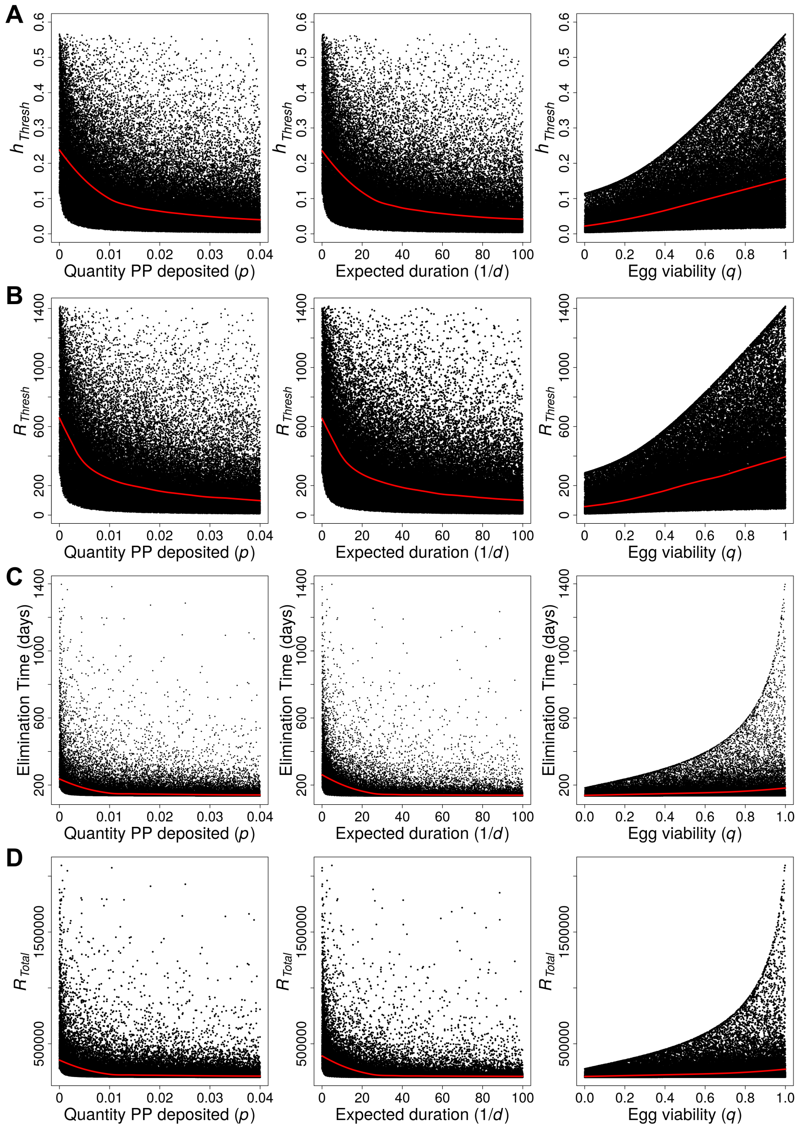

Supplementary Figure 4: Sensitivity analysis with fixed release rate and competitiveness. Results from sensitivity analyses of the BSIT model with competitiveness $h = 0.2$ (B,C,D) and daily release rate $R = 500$ (A) or $R = 1500$ (C,D). The expected value (red lines) of the threshold h_{Thresh} was most sensitive to the quantity of pyriproxifen deposited by females (p) and the longevity of pyriproxifen in the environment ($1/d$), whereas the variance in h_{Thresh} was most sensitive to egg viability (q) (A). Similar patterns in the sensitivity to each parameter are observed in R_{Thresh} (B), elimination time (C), and total release required for elimination (D). One hundred thousand parameter randomisations are used per row. Rows C and D use the same set of parameter randomisations.

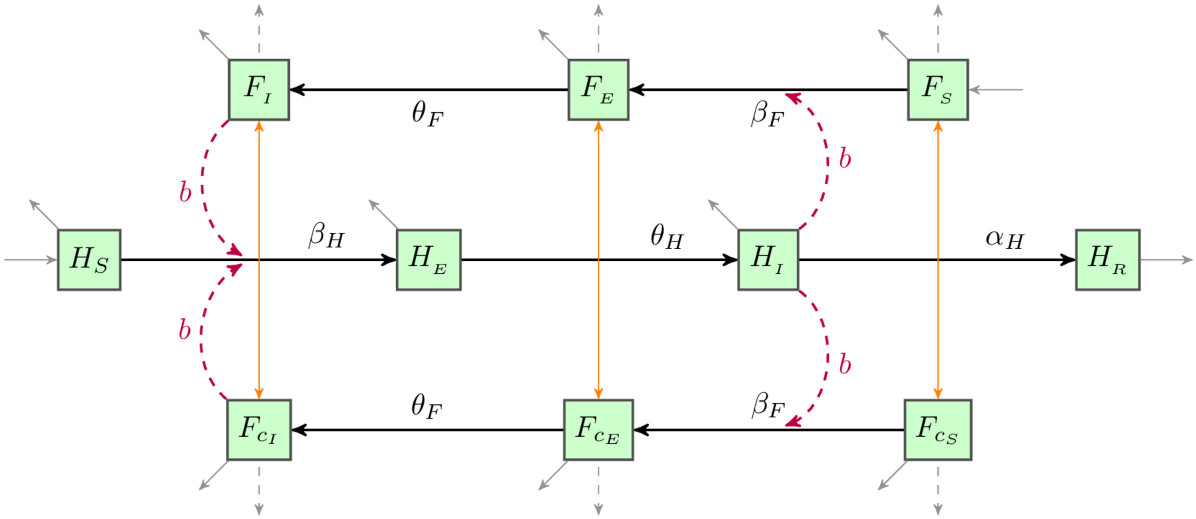

Supplementary Figure 5: Incorporation of dengue transmission within the BSIT model. Humans are categorised susceptible (H_S), exposed (H_E), infectious (H_I) or recovered (H_R). Female mosquitoes are characterised as susceptible (F_S), exposed (F_E) or infectious (F_I). Suffix c indicates pyriproxifen contamination. Parameters include bite rate (b), transition probabilities (β_F, β_H) and transmission rates (θ_F,θ_H,α_H).

Supplementary Tables

Parameter	Definition	Fixed Value	Range	References
N	Number of larvae sites (per hectare)	200	5–200	(*1–5*)
K_1	Carrying capacity of larvae at one larval site	25	15–60	(*6–8*)
V_1	Volume of water larvae at one larval site (litres)	0.25	0.1–1.0	(*6–8*)
g^{-1}	Expected gonotrophic cycle length	4	2.5–10	(*9*)
f	Fecundity per gonotrophic cycle at low density	60	30–100	(*9–12*)
ρ	Proportion of females among juveniles	0.4	0.3–0.5	(*9, 13*)
m_E^{-1}	Maturation time of eggs	4	2–7	(*9, 14, 15*)
m_L^{-1}	Maturation time of larvae (days)	8	6–12	(*9, 14*)
m_P^{-1}	Maturation time of pupae (days)	3	2–5	(*9, 16*)
μ_E	Mortality rate of eggs	0.05	0.01–0.25	(*17*)
μ_0^{-1}	Baseline life expectancy of larvae	35	25–45	(*9*)
μ_K	Mortality rate of larvae at carrying capacity	$\frac{m_E m_L m_P \rho g f}{(m_E+\mu_E)(m_P+\mu_P)\mu_F} - m_L$	–	–
μ_P	Mortality rate of pupae	0.06	0.01–0.17	(*9, 16, 18*)
μ_F^{-1}	Life expectancy of adult females	15.5	13.8–24.5	(*19, 20*)
μ_M^{-1}	Life expectancy of adult males	13.6	12.8–24.5	(*19, 20*)
μ_S^{-1}	Life expectancy of sterile males	11.6	5.5–20.9	(*19, 20*)
μ_c	Mortality rate of contaminated adult females	μ_F	–	(*21, 22*)
κ	Number of ovipositions per gonotrophic cycle	2	1–12	(*23–28*)
κ_c	Expected number of contaminating ovipositions	1	Unknown	
γ	Oviposition rate	κg	–	–
r	Coupling rate of an adult male	0.94	0.5–1.2	(*29*)
h	Ratio of sterile male / natural male coupling rates	0.2	0–1	(*30–32*)
p	Mass of pyriproxifen deposited at oviposition	0.008	0.002–0.04	(*21, 33*)
q	Relative viability of eggs from contaminated females	0.51	0.4–0.6	(*34*)
d^{-1}	Expected duration of contamination at larval sites	33	5–100	(*35, 36*)
EI_{50}	Pyriproxifen concentration for 50% emergence inhibition	0.2	0.168–0.229	(*21*)
EI_{95}	Pyriproxifen concentration for 95% emergence inhibition	0.668	0.547–0.902	(*21*)
σ	Slope of the dose-response curve	$\frac{\text{logit}(0.95)-\text{logit}(0.5)}{\ln(EI_{95})-\ln(EI_{50})}$	–	–
A	Number of auto-dissemination stations / ha	–	–	See Sup. Table 3
α	Contamination rate at one dissemination station	–	–	See Sup. Table 3

Supplementary Table S1. Parameters of the BSIT/SIT model, including default (fixed) values, ranges found in the literature and references of source publications.

Parameter	Symbol	Value	Alternative value	References
Bite rate	b	0.5	0.26	(*37, 38*)
Mosquito to human transmission prob.	β_H	0.75	0.31	(*37, 38*)
Human to mosquito transmission prob.	β_F	0.75	0.31	(*37, 38*)
Intrinsic incubation period	$1/\theta_H$	5.9	-	(*39*)
Extrinsic incubation period	$1/\theta_F$	8.3	10.0	(*37*)
Recovery rate in humans	α_H	1/6	-	(*38*)
Human death rate	μ_H	1/(72×365.25)	-	(*40*)
Human population density	H	50	-	(*41*)

Supplementary Table S2. Parameters of the dengue transmission model. Values of four parameters in the "Alternative value" column are taken from (*38*) and provide a relatively "optimistic" R_0 compared to the more "pessimistic" values taken from (*37*). The human mortality rate is set to the inverse of the 2016 global mean life expectancy. The chosen human population density is approximately equivalent to that of Seville or Montpellier.

Paper	Reference	Stations	Area	St/ha	Time	EI	α
Caputo (2012)	(*42*)	10	1 ha	10	20 d	51.6%	0.0035
Abad-Franch (2015)	(*43*)	100	7 ha	14	121 d	63.3%	0.09
Chandel (2016)	(*44*)	32	1.6 ha	20	14 d	38.8%	0.0019
Unlu (2017)	(*45*)	75	3.8 ha	19.8	49 d	69.4%	0.0024
Abad-Franch (2017)	(*46*)	1000	650 ha	1.54	152 d	79.3%	2.9500

Supplementary Table S3. Data from five auto-dissemination field trials and associated estimates of contamination rate at dissemination station (α). Trajectories of the ADT model were simulated with dissemination station density (A) set to St/ha. Calibration of α involved minimising the absolute error between modelled and observed emergence inhibition (EI) at a given point in time (Time). Calibration assumed EI in ovitraps equalled EI at larval sites. *Aedes albopictus* was present in all five studies. *Aedes aegypi* was present only in the two Brazilian (Abad-Franch) trials.

Supplementary References

1. Morrison, A. C. et al. Evaluation of a sampling methodology for the rapid assessment of *Aedes aegypti* infestation levels in Iquitos, Perú. *Revista Peruana de Epidemiología* **10**, 1 (2002).

2. Silver, J. B. *Mosquito ecology: field sampling methods*. 3rd ed. (Springer Science & Business Media, New York, 2007).

3. Favaro, E. A., Dibo, M. R., Pereira, M., Chierotti, A. P., Rodrigues-Junior, A. L. & Chiaravalloti-Neto, F. *Aedes aegypti* entomological indices in an endemic area for dengue in Sao Paulo State, Brazil. *Rev. Saude Publ.* **47**, 588–597 (2013).

4. de Brito Arduino, M. Assessment of *Aedes aegypti* pupal productivity during the dengue vector control program in a costal urban centre of São Paulo State, Brazil. *J. Insects* **2014**, 1–9 (2014).

5. Maciel-de Freitas, R., Marques, W. A., Peres, R. C., Cunha, S. P. & Loureno-de Oliveira, R. Variation in *Aedes aegypti*(Diptera: Culicidae) container productivity in a slum and a suburban district of Rio de Janeiro during dry and wet seasons. *Mem. Inst. Oswaldo Cruz* **102**, 489–496 (2014).

6. Ramasamy, R., Surendran, S. N., Jude, P. J., Dharshini, S. & Vinobaba, M. Larval development of *Aedes aegypti* and *Aedes albopictus* in peri-urban brackish water and its implications for transmission of arboviral diseases. *PLoS Neglect. Trop. D.* **5**, e1369 (2011).

7. Bartlett-Healy, K. et al. Larval mosquito habitat utilization and community dynamics of *Aedes albopictus* and *Aedes japonicus* (Diptera: Culicidae). *J. Med. Entomol.* **49**, 813–824 (2012).

8. Li, Y. J. et al. Urbanization increases *Aedes albopictus* larval habitats and accelerates mosquito development and survivorship. *PLoS Neglect. Trop. D.* **8**, 1–12 (2014).

9. Delatte, H., Gimonneau, G., Triboire, A. & Fontenille, D. Influence of temperature on immature development, survival, longevity, fecundity, and gonotrophic cycles of *Aedes albopictus*, vector of Chikungunya and dengue in the Indian Ocean. *J. Med. Entomol.* **46**, 33–41 (2009).

10. Blackmore, M. S., Lord, C. C. et al. The relationship between size and fecundity in *Aedes albopictus*. *J. Vector Ecol.* **25**, 212–217 (2000).

11. Maciá, A. Differences in performance of *Aedes aegypti* larvae raised at different densities in tires and ovitraps under field conditions in Argentina. *J. Vector Ecol.* **31**, 371–377 (2006).

12. Panigrahi, S. K., Barik, T. K., Mohanty, S. & Tripathy, N. K. Laboratory evaluation of oviposition behavior of field collected *Aedes* mosquitoes. *J. Insects* **2014**, 1–8 (2014).

13. Christophers, S. & *et al.* Aedes aegypti *(L.) the yellow fever mosquito: its life history, bionomics and structure.* (Cambridge University Press, New York, 1960).

14. Focks, D. A., Haile, D., Daniels, E. & Mount, G. A. Dynamic life table model for *Aedes aegypti* (Diptera: Culicidae): analysis of the literature and model development. *J. Med. Entomol.* **30**, 1003–1017 (1993).

15. Monteiro, L. C. C., de Souza, J. R. & de Albuquerque, C. M. R. Eclosion rate, development and survivorship of *Aedes albopictus* (Skuse)(Diptera: Culicidae) under different water temperatures. *Neotrop. Entomol.* **36**, 966–971 (2007).

16. Lee, S. J. Development of eggs, larvae and pupae of *Aedes albopictus* (Skuse) (Diptera: Culicidae). *Chin. J. Entomol.* **14**, 13–32 (1994).

17. Tran, A. et al. A rainfall-and temperature-driven abundance model for *Aedes albopictus* populations. *Int. J. Environ. Res. Public Health* **10**, 1698–1719 (2013).

18. Castro Gomes, A. d., Gotlieb, S. L. D., Marques, C. C., Paula, M. B. d. & Marques, G. R. A. M. Duration of larval and pupal development stages of *Aedes albopictus* in natural and artificial containers. *Rev. Saude Publ.* **29**, 15–19 (1995).

19. Oliva, C. F. et al. Effects of irradiation, presence of females, and sugar supply on the longevity of sterile males *Aedes albopictus* (Skuse) under semi-field conditions on Reunion Island. *Acta Trop.* **125**, 287–293 (2013).

20. Lacroix, R., Delatte, H., Hue, T. & Reiter, P. Dispersal and survival of male and female *Aedes albopictus* (Diptera: Culicidae) on Reunion Island. *J. Med. Entomol.* **46**, 1117–1124 (2009).

21. Dell Chism, B. & Apperson, C. S. Horizontal transfer of the insect growth regulator pyriproxyfen to larval microcosms by gravid *Aedes albopictus* and *Ochlerotatus triseriatus* mosquitoes in the laboratory. *Med. Vet. Entomol.* **17**, 211–220 (2003).

22. Mains, J. W., Brelsfoard, C. L. & Dobson, S. L. Male mosquitoes as vehicles for insecticide. *PLoS Neglect. Trop. D.* **9**, e0003406–e0003406 (2015).

23. Reiter, P., Amador, M. A., Anderson, R. A. & Clark, G. G. Short report: Dispersal of *Aedes aegypti* in an urban area after blood feeding as demonstrated by rubidium-marked eggs. *Am. J. Trop. Med. Hyg.* **52**, 177–179 (1995).

24. Trexler, J. D., Apperson, C. S. & Schal, C. Laboratory and field evaluations of oviposition responses of *Aedes albopictus* and *Aedes triseriatus* (Diptera: Culicidae) to oak leaf infusions. *J. Med. Entomol.* **35**, 967–976 (1998).

25. Harrington, L. C. & Edman, J. D. Indirect evidence against delayed "skip-oviposition" behavior by *Aedes aegypti* (Diptera: Culicidae) in Thailand. *J. Med. Entomol.* **38**, 641–645 (2001).

26. Trexler, J. D. et al. Role of bacteria in mediating the oviposition responses of *Aedes albopictus* (Diptera: Culicidae). *J. Med. Entomol.* **40**, 841–848 (2003).

27. Colton, Y., Chadee, D. & Severson, D. Natural skip oviposition of the mosquito *Aedes aegypti* indicated by codominant genetic markers. *Med. Vet. Entomol.* **17**, 195–204 (2003).

28. Ponnusamy, L., Xu, N., Nojima, S., Wesson, D. M., Schal, C. & Apperson, C. S. Identification of bacteria and bacteria-associated chemical cues that mediate oviposition site preferences by *Aedes aegypti*. *Proc. Natl. Acad. Sci.* **105**, 9262–9267 (2008).

29. Boyer, S., Gilles, J., Merancienne, D., Lemperiere, G. & Fontenille, D. Sexual performance of male mosquito *Aedes albopictus*. *Med. Vet. Entomol.* **25**, 454–459 (2011).

30. Oliva, C. F. et al. The sterile insect technique for controlling populations of *Aedes albopictus* (Diptera: Culicidae) on Reunion Island: mating vigour of sterilized males. *PLoS One* **7**, e49414–e49414 (2012).

31. Bellini, R., Balestrino, F., Medici, A., Gentile, G., Veronesi, R. & Carrieri, M. Mating competitiveness of *Aedes albopictus* radio-sterilized males in large enclosures exposed to natural conditions. *J. Med. Entomol.* **50**, 94–102 (2013).

32. Madakacherry, O., Lees, R. S. & Gilles, J. R. L. *Aedes albopictus* (Skuse) males in laboratory and semi-field cages: release ratios and mating competitiveness. *Acta Trop.* **132**, S124–S129 (2014).

33. Gaugler, R., Suman, D. & Wang, Y. An autodissemination station for the transfer of an insect growth regulator to mosquito oviposition sites. *Med. Vet. Entomol.* **26**, 37–45 (2012).

34. Primault, L. *Comment booster la technique de l'insecte stérile? Transfert de pyriproxyfène par les mâles aux femelles et impact sur leur reproduction.* (Master's thesis, Université de Montpellier, 2015).

35. World Health Organization. *WHO specifications and evaluations for public health pesticides: pyriproxyfen*. (World Health Organization, Geneva, Switzerland, 2006).

36. Sullivan, J. J. & Goh, K. S. Environmental fate and properties of pyriproxyfen. *J. Pestic. Sci.* **33**, 339–350 (2008).

37. de Pinho, S. T. R., Ferreira, C. P., Esteva, L., Barreto, F. R., e Silva, V. C. M. & Teixeira, M. G. L. Modelling the dynamics of dengue real epidemics. *Philos. T. R. Soc. A.* **368**, 5679–5693 (2010).

38. Manore, C. A., Hickmann, K. S., Xu, S., Wearing, H. J. & Hyman, J. M. Comparing dengue and Chikungunya emergence and endemic transmission in *Ae. aegypti* and *Ae. albopictus*. *J. Theor. Biol.* **356**, 174–191 (2014).

39. Chan, M. & Johansson, M. A. The incubation periods of dengue viruses. *PLoS One* **7**, e50972 (2012).

40. World Health Organisation. *WHO methods and data sources for life tables 1990-2016*. Geneva, (2018).

41. Wikipedia contributors. List of European Union cities proper by population density. *Wikipedia, The Free Encyclopedia.* `https://en.wikipedia.org/wiki/List\_of\_European\_Union\_cities\_proper\_by\_population\_density`. Accessed: 2019-13-0.3.

42. Caputo, B. et al. The auto-dissemination approach: a novel concept to fight *Aedes albopictus* in urban areas. *PLoS Neglect. Trop. D.* **6**, e1793 (2012).

43. Abad-Franch, F., Zamora-Perea, E., Ferraz, G., Padilla-Torres, S. D. & Luz, S. L. B. Mosquito-disseminated pyriproxyfen yields high breeding-site coverage and boosts juvenile mosquito mortality at the neighborhood scale. *PLoS Neglect. Trop. D.* **9**, e0003702 (2015).

44. Chandel, K. et al. Targeting a hidden enemy: pyriproxyfen autodissemination strategy for the control of the container mosquito *Aedes albopictus* in cryptic habitats. *PLoS Neglect. Trop. D.* **10**, e0005235 (2016).

45. Unlu, I., Suman, D. S., Wang, Y., Klingler, K., Faraji, A. & Gaugler, R. Effectiveness of autodissemination stations containing pyriproxyfen in reducing immature *Aedes albopictus* populations. *Parasit. Vectors* **10**, 139 (2017).

46. Abad-Franch, F., Zamora-Perea, E. & Luz, S. L. Mosquito-disseminated insecticide for citywide vector control and its potential to block arbovirus epidemics: Entomological observations and modeling results from Amazonian Brazil. *PLoS Med.* **14**, e1002213 (2017).
